# The Cut-Off Point and Boundary Values of Waist-to-Height Ratio as an Indicator for Cardiovascular Risk Factors in Chinese Adults from the PURE Study

**DOI:** 10.1371/journal.pone.0144539

**Published:** 2015-12-07

**Authors:** Yaguang Peng, Wei Li, Yang Wang, Jian Bo, Hui Chen

**Affiliations:** State Key Laboratory of Cardiovascular Disease, Fuwai Hospital, National Center for Cardiovascular Disease, Chinese Academy of Medical Sciences and Peking Union Medical College, Beijing, China; McMaster University, CANADA

## Abstract

To explore a scientific boundary of WHtR to evaluate central obesity and CVD risk factors in a Chinese adult population. The data are from the Prospective Urban Rural Epidemiology (PURE) China study that was conducted from 2005–2007. The final study sample consisted of 43 841 participants (18 019 men and 25 822 women) aged 35–70 years. According to the group of CVD risk factors proposed by Joint National Committee 7 version and the clustering of risk factors, some diagnosis parameters, such as sensitivity, specificity and receiver operating characteristic (ROC) curve least distance were calculated for hypertension, diabetes, high serum triglyceride (TG), high serum low density lipoprotein cholesterol (LDL-C), low serum high density lipoprotein cholesterol (HDL-C) and clustering of risk factors (number≥2) to evaluate the efficacy at each value of the WHtR cut-off point. The upper boundary value for severity was fixed on the point where the specificity was above 90%. The lower boundary value, which indicated above underweight, was determined by the percentile distribution of WHtR, specifically the 5^th^ percentile (P_5_) for both males and females population. Then, based on convenience and practical use, the optimal boundary values of WHtR for underweight and obvious central obesity were determined. For the whole study population, the optimal WHtR cut-off point for the CVD risk factor cluster was 0.50. The cut-off points for severe central obesity were 0.57 in the whole population. The upper boundary values of WHtR to detect the risk factor cluster with specificity above 90% were 0.55 and 0.58 for men and women, respectively. Additionally, the cut-off points of WHtR for each of four cardiovascular risk factors with specificity above 90% in males ranged from 0.55 to 0.56, whereas in females, it ranged from 0.57 to 0.58. The P_5_ of WHtR, which represents the lower boundary values of WHtR that indicates above underweight, was 0.40 in the whole population. WHtR 0.50 was an optimal cut-off point for evaluating CVD risks in Chinese adults of both genders. The optimal boundaries of WHtR were 0.40 and 0.57, indicating low body weight and severe risk for CVD, respectively, in Chinese adults.

## Introduction

Central obesity has been a growing worldwide health problem [1]. As has been reported, it is one of the well-known risk factors for cardiovascular disease (CVD) and is shown to be associated with hypertension, diabetes mellitus and dyslipidemia [2–6]. Waist circumference (WC), which could be easily measured in population-based epidemiologic studies, is one of the indices for central obesity often used worldwide. However, WC, with gender-specific and ethnic differences, is correlated with body-frame size; thus, the efficacy of WC for central obesity diagnosis and prediction is diminished, especially for tall or short individuals [7, 8]. In 1995 and 1996, waist-to-height ratio (WHtR) was, for the first time, referred to as an anthropometric measure by researchers in Japan and the UK [9, 10], who also suggested that the same cut-off point value (WHtR 0.50) for central obesity and CVD risks be used in both men and women [11, 12]. Furthermore, in practical use without evidence, the WHtR boundary values of 0.40 and 0.60 were introduced to indicate underweight and severe obesity risk, respectively [13–15]. WHtR 0.50 can be the cut-off point for central obesity and CVD risks with no gender-specific, ethnicity-specific or height-corrected advantages. However, this point value alone cannot show the severity and risks of obesity. Additionally, this does not imply that lower WHtR is necessarily better. Thus, reasonable boundary values of WHtR are needed to be scientifically certain in order to differentiate severity level for better weight control and CVD prevention. The purpose of this study was to explore a scientific boundary of WHtR using data from the PURE—China study, whose samples from urban and rural area in China, to evaluate central obesity and CVD risk factors in Chinese adults.

## Methods

The methods used to conduct this study were consistent with the PURE study, and have been reported previously [16, 17]. The PURE—China study was approved by the ethics committees of the National Center for Cardiovascular Diseases in China and details are summarized below.

### Samples

Prospective Urban Rural Epidemiology (PURE) was an international multi-center prospective study. More than 600 communities and over 140 000 participants were enrolled in this study based on the countries’ income levels, which were categorized as low-, middle- and high-income. The baseline data for the China cohort in the PURE study were used for this analysis. A total of 46 285 participants aged 35–70 from 12 centers (Yunnan, Qinghai, Beijing, Nanjing, Shandong, Shanxi, Shaanxi, Liaoning, Nanchang, Inner Mongolia, Xinjiang and Sichuan) in mainland China were screened for cardiovascular diseases risk factors in the PURE-China study conducted from 2005–2007. The participants in PURE were selected in a three-stage sampling process; first by community, then by household, and finally by individual in a household. All eligible individuals (35–70 years old) in the selected households who provided written informed consent were enrolled. Data, including demographic information and physical and laboratory examination, were collected in the baseline survey. We have excluded participants with incomplete data for the standardized physical measures and laboratory examination. In addition, pregnant women were excluded. The final study sample consisted of 43 841 participants (18 019 men and 25 822 women).

### Survey Methods

We gathered information about demographics, physical activity level, diet, smoking and other risk factors using a uniform questionnaire for adult participants, following the same procedures and methods used in studies in the other international centers. Quality of data collection was maintained through the use of standardized protocols and centralized training. The investigators and supervisors were jointly trained in standardized field methods, based on the PURE protocol and a manual of operations.

### Physical Examination

Participants were measured in casual clothing without shoes in a relaxed standing position. Height was measured in meters (m) to the nearest centimeter using a standard right angle device and body weight was measured in kilograms (kg) by a spring balance to the nearest kilogram. Waist circumference (WC) was measured to the nearest centimeter using a tape measure at the high point of the iliac crest at minimal respiration when the participants were in standing position. Waist-to-height ratio (WHtR) was calculated as the ratio of waist (cm) to height (cm). To minimize the errors in measurement because of body position or in reading and recording the measurements, study staff members were trained in the standardized protocols. One study staff member assisted a second study staff member in positioning the participants and in reading the measurements to prevent errors.

For all participants, blood pressure was taken three times in the right arm at each session using a standard mercury sphygmomanometer. Participants were seated comfortably and had rested at least 5 minutes, had an empty bladder and had not smoked for at least 15 minutes. Proper placement of cuff and stethoscope, cuff inflation 20 mmHg higher than radial pulse obliteration, rapid cuff inflation and a constant deflation rate of 2 mmHg/s were emphasized. SBP was measured at Korotkoff phase I and DBP as Korotkoff phase V [16, 17]. The mean of the three measurements was used in the analysis.

### Laboratory Measurements

A 12-hour fasting blood sample was collected, including fasting blood glucose (FBG), serum triglycerides (TG), serum low density lipoprotein cholesterol (LDL-C), and serum high density lipoprotein cholesterol (HDL-C) levels, and measured in a centralized, certified laboratory. Staff at the local centers were trained and certified by each center. The 12-hour FBG was measured by an enzymatic method (Company). TG were measured with the Boehringer-Mannheim Diagnostics High-Performance enzymatic reagent (Boehringer-Mannheim, Indianapolis, Indiana, USA) on the Abbott Laboratories ABA 200 bichromatic analyzer (Abbott, Chicago, Illinois, USA). Serum HDL-C level was measured by the same enzymatic method used for serum cholesterol after precipitation of other lipoprotein fractions using 50 000 molecular weight dextran sulfate with Mg^2+^ (Boehringer-Mannheim, Indianapolis, Indiana, USA). A free glycerol blank correction was made on all specimens.

### Definition of CVD Risk Factors

In the present study, we adopted the definition of CVD risk factors proposed by the Seventh Report of the Joint National Committee (JNC7) [18]. The criteria for hypertension diagnosis were defined as follows: systolic blood pressure ≥140 mmHg and/or diastolic blood pressure ≥ 90 mmHg and/or current use of antihypertensive medication. Participants with FBG ≥ 7.0 mmol/L and/or current use of medication or insulin were defined as having diabetes. High LDL-C was defined as LDL-C ≥ 3.12 mmol/L(120 mg/dL) or use of medication to lower; high TG was defined as TG ≥ 1.70 mmol/L (150 mg/dL) or use of medication to lower; and low HDL-C was defined as HDL-C < 1.04 mmol/L (40 mg/dL). We defined a risk factor cluster as one individual having any 2 or more of the following risk factors: hypertension, diabetes and dyslipidemia (high LDL-C, high TG and low HDL-C).

### Statistical Analysis

Diagnostic indices, including sensitivity (Sen) and specificity (Spe), were calculated for hypertension, diabetes mellitus, high TG, high LDL-C, low HDL-C and clustering of risk factors (number ≥ 2) to evaluate the efficacy of each point of WHtR, ranging from 0.48 to 0.60 by 0.01 step-length. The receiver operating characteristic (ROC) curve least distance was calculated as the linear distance between the optimal point and the (0,1) point in the coordinate to determine the cut-off point values of WHtR for evaluating risk factors of CVD. The boundary values for severe risks were fixed on the points where specificity was above 90%. The boundary values used to indicate underweight were determined to be the 5^th^ percentile (P_5_) of the point for both males and females, based on the percentile distribution of WHtR. Then, based on convenience and practical use, the optimal cut-off points of WHtR for CVD risk and underweight in the whole population were determined.

Continuous variables are presented as the mean±standard deviation (SD), and categorical variables are presented as cases (n) or percentage (%). Comparisons between groups were performed using Student’s 2-tailed t-test for continuous variables and chi-square test for categorical data. A value of *P* < 0.05 was considered statistically significant. SPSS 20.0 statistical software package (SPSS & IBM, Inc., Chicago, Illinois, USA) was used to perform statistical analysis.

## Results

### Participant Characteristics

The characteristics of the study participants and the prevalence of CVD risk factors, for the entire study population and stratified by gender, are presented in **Table 1**. The mean age of males and females was 51.7 ± 9.9 years and 51.0 ± 9.6 years, respectively. The prevalence rates among male participants of hypertension, high TG, and risk factor clusters was significantly higher than the corresponding prevalence rates among female participants, and the rates of females with high LDL-C and low HDL-C were higher than those of males. The mean values of WHtR were significantly different between genders. However, the absolute difference was small.

**Table 1:** Characteristics of the Study Chinese Population in the PURE Study.

	All Subjects	Male	Female
**Number(cases)**	43841	18019	25822
**Age(year)**	51.27±7.17	51.68±9.91	50.98±9.62
**Height(cm)**	160.79±8.25	167.42±6.48	156.15±5.83†
**Weight(kg)**	63.82±12.13	68.76±12.10	60.37±10.89†
**WC(cm)**	81.10±10.54	83.76±10.40	79.24±10.24†
**WHtR**	0.50±0.06	0.51±0.06	0.50±0.06*
**BMI(m/kg** ^**2**^ **)**	24.63±4.02	24.48±3.80	24.74±4.15*
**SBP(mmHg)**	133.56±22.42	135.08±21.09	132.51±23.25†
**DBP(mmHg)**	82.70±12.25	83.87±12.38	81.88±12.09*
**FBG(mmol/L)**	5.56±1.56	5.56±1.55	5.57±1.56
**TG(mmol/L)**	1.55±1.10	1.60±1.20	1.51±1.02
**LDL-C(mmol/L)**	2.63±0.79	2.56±0.76	2.67±0.81†
**HDL-C(mmol/L)**	1.36±0.33	1.32±0.33	1.39±0.33†
**HP(%)**	42.7	45.3	40.9†
**DM(%)**	9.2	9.3	9.1
**High TG(%)**	29.6	31.0	28.6*
**High LDL-C(%)**	23.4	20.6	25.4†
**Low HDL-C(%)**	17.9	12.2	14.6†
**RFC(%)**	35.4	37.1	34.2†

Values are means ± SD or n or percentages (%).

* P<0.05.

†P<0.001

* or †, Values of female group are significantly different from those of male group.

WC, waist circumference; WHtR, waist-to-height ratio; SBP, systolic blood pressure; DBP, diastolic blood pressure; FBG, fasting blood glucose; TG, triglycerides; LDL-C, low-density lipoprotein cholesterol; HDL-C, high-density lipoprotein cholesterol; HP, hypertension; DM, diabetes mellitus; RFC, risk factors cluster.

### Cut-off Point Values of WHtR for CVD Risk Factor Clusters

The ranked cut-off points of WHtR for CVD risk factor clusters ranging from 0.48–0.60 in increments of 0.01 in the whole study population and among male and female participants are shown in **Table 2**. The optimal cut-off point values for risk factor clusters was 0.50 in males and 0.51 in females, based on the ROC least distance. For the whole study population, the optimal WHtR cut-off point value was 0.50. The cut-off points of WHtR, whose specificity for evaluating the clustering of risk factors was over 90.0%, were also listed for the whole study population, males and females. These indicate the severity of CVD risk and were 0.57 in the whole population, 0.56 among males and 0.57 among females.

**Table 2:** Cut Points of WHtR for Predictive of CVD Risk Factor Clusters and Severity Central Obesity.

	Value	Sen.	Spe.	ROC Least Dis.
**All Subjects (n = 43841)**	0.48	0.791	0.489	0.552
	0.49	0.736	0.554	0.518
	0.50	0.675	0.618	0.501
	0.51	0.610	0.680	0.505
	0.52	0.539	0.735	0.531
	0.53	0.467	0.785	0.575
	0.54	0.399	0.826	0.626
	0.55	0.333	0.862	0.681
	0.56	0.275	0.893	0.733
	0.57	0.226	0.917	0.778
	0.58	0.181	0.935	0.821
	0.59	0.142	0.950	0.860
	0.60	0.111	0.963	0.890
**Male (n = 18019)**	0.48	0.790	0.504	0.538
	0.49	0.728	0.569	0.510
	0.50	0.657	0.635	0.500
	0.51	0.583	0.701	0.513
	0.52	0.502	0.759	0.554
	0.53	0.422	0.810	0.608
	0.54	0.350	0.850	0.667
	0.55	0.282	0.886	0.727
	0.56	0.227	0.916	0.777
	0.57	0.179	0.939	0.823
	0.58	0.134	0.955	0.867
	0.59	0.097	0.966	0.903
	0.60	0.070	0.975	0.930
**Female (n = 25822)**	0.48	0.792	0.480	0.560
	0.49	0.742	0.545	0.524
	0.50	0.689	0.607	0.501
	0.51	0.630	0.666	0.499
	0.52	0.568	0.720	0.515
	0.53	0.501	0.768	0.550
	0.54	0.436	0.810	0.595
	0.55	0.371	0.846	0.647
	0.56	0.311	0.877	0.700
	0.57	0.262	0.902	0.745
	0.58	0.217	0.922	0.787
	0.59	0.176	0.939	0.826
	0.60	0.142	0.954	0.859

Values are cut-off points of WHtR in the first column, ROC least distances in the last column in the other columns, which indicated some main diagnostic rate.

Sen, sensitivity; Spe, specificity; ROC Least Dis, receiver operating characteristic least distance.

$$\text{ROC}\;\text{Least}\;\text{Dis}.=\sqrt{\;(1\;\text{-}\;\text{Spe}{)}^{2}+{\left(1\;\text{-}\;\text{Sen}\right)}^{2}}$$

### Cut-off Point Values of WHtR for Evaluation of Each CVD Risk Factor

The cut-off point values of WHtR for evaluating each single CVD risk factor, including hypertension, diabetes, high TG, high LDL-C and low HDL-C, were also calculated and are listed in **Table 3**. The cut-off point value was found to be at 0.50 for males in each individual risk factor and ranged from 0.50 to 0.52 in females. Furthermore, it ranged from 0.50 to 0.51 for the whole study population as well. Diagnosis parameters for each risk at each point, including sensitivity and specificity, are listed in the online appendix table (**S1–S5 Tables**).

**Table 3:** Optimal Cut-off Point Values of WHtR for Evaluation of CVD Risk Factors.

	Value	Sen.	Spe.	ROC Least Dis.
**Hypertension**				
**ALL**	0.50	0.625	0.619	0.535
**Male**	0.50	0.589	0.623	0.558
**Female**	0.50	0.653	0.616	0.518
**Diabetes**				
**ALL**	0.51	0.640	0.599	0.539
**Male**	0.50	0.669	0.547	0.561
**Female**	0.52	0.616	0.645	0.523
**High TG**				
**ALL**	0.50	0.680	0.596	0.515
**Male**	0.50	0.673	0.617	0.504
**Female**	0.51	0.624	0.640	0.520
**High LDL-C**				
**ALL**	0.50	0.557	0.536	0.642
**Male**	0.50	0.548	0.546	0.640
**Female**	0.50	0.561	0.529	0.644
**Low HDL-C**				
**ALL**	0.50	0.514	0.519	0.684
**Male**	0.50	0.531	0.539	0.657
**Female**	0.50	0.496	0.506	0.705

Values are cut-off points of WHtR in the first column, ROC least distances in the last column and percentage rates (%) in the other columns, which indicated some main diagnostic rate.

Abbreviations see Tables 1 and 2.

### Boundary Values of WHtR for Severity Level of each CVD Risk Factor

All of the boundary values for severe risk of CVD were assessed for each single CVD risk factor and are shown in **Table 4**. The values of WHtR for severe central obesity ranged from 0.56 to 0.57 in males, 0.57 to 0.59 in females and 0.57 to 0.59 in the whole population. All other diagnosis parameters for severity levels at specificity above 90% are listed in the online appendix tables (**S1–S5 Tables**).

**Table 4:** Boundary Values of WHtR for Severity of Central Obesity in Each CVD Risk Factors.

	Value	Sen.	Spe.	ROC Least Dis.
**Hypertension**				
**ALL**	0.57	0.207	0.921	0.797
**Male**	0.56	0.201	0.915	0.803
**Female**	0.57	0.245	0.909	0.760
**Diabetes**				
**ALL**	0.58	0.207	0.904	0.799
**Male**	0.57	0.193	0.904	0.822
**Female**	0.59	0.201	0.910	0.804
**High TG**				
**ALL**	0.57	0.225	0.905	0.781
**Male**	0.56	0.229	0.904	0.777
**Female**	0.58	0.212	0.909	0.793
**High LDL-C**				
**ALL**	0.58	0.125	0.800	0.881
**Male**	0.57	0.123	0.900	0.883
**Female**	0.59	0.114	0.905	0.891
**Low HDL-C**				
**ALL**	0.59	0.081	0.917	0.923
**Male**	0.57	0.127	0.900	0.879
**Female**	0.60	0.077	0.921	0.926

Values are cut-off points of WHtR in the first column, ROC least distances in the last column and percentage rates (%) in the other columns, which indicated some main diagnostic rate.

Abbreviations see Tables 1 and 2.

### Percentile Distribution of WHtR and Boundary Values of WHtR for Underweight

The percentile distribution of WHtR, stratified by gender, is shown in **Table 5**. The median (P_50_) of WHtR was 0.50. Furthermore, the P_5_, which could indicate underweight, was 0.40 in both males and females. P_90_, which could be defined to be severe central obesity, was 0.58 in males and 0.60 in females. These two percentiles, P_5_ and P_90_, were 0.40 and 0.59 in the whole population, respectively. The P_5_ value of WHtR (0.40) was considered the indicator for above underweight.

**Table 5:** Percentile Distribution of WHtR of Study Population for Indicating Underweight.

Percentile	Total (n = 43 841)	Male (n = 18 019)	Female (n = 25 822)
**P**_**1**_	0.37	0.37	0.37
**P**_**2.5**_	0.39	0.39	0.39
**P**_**5**_	0.41	0.40	0.41
**P**_**10**_	0.42	0.42	0.43
**P**_**25**_	0.46	0.46	0.46
**P**_**50**_	0.50	0.50	0.50
**P**_**75**_	0.55	0.54	0.55
**P**_**90**_	0.59	0.58	0.60
**P**_**95**_	0.61	0.60	0.62
**P**_**97.5**_	0.64	0.62	0.65
**P**_**99**_	0.67	0.64	0.68

Values are points of WHtR in percentile, indicated low level of weight.

P_1_, 1^st^ percentile, etc.

## Discussion

Based on the results of this study, we determined that the cut-off point for WHtR of 0.50, which took into account body size, could be an effective measure for central obesity and CVD risk in the Chinese adult population. Recently, some studies thought that WC could be used as an indicator of risk, independent of height [19–22]. However, an increasing number of studies have provided evidence that WHtR could be useful in predicting central obesity and CVD risk because its correlation to height could result in a bias when using WC alone. The cut-off point of WHtR for central obesity of 0.50 is recommended in both child and adult populations in studies worldwide [23–32]. A systematic review [12] also supported the cut-off point of WHtR at 0.50 and sent a message that, to protect their health, individuals should ‘keep their waist size at half of their height’.

In our study, as expected, we found the optimal cut-off point of WHtR for CVD risk factor clusters in the whole population was 0.50. In **Table 1**, we presented the mean value of WHtR in females, which was equal to the cut-off point of 0.50, whereas the mean value in males was 0.49. In **Table 5**, the gender-stratified medians of WHtR are also shown to be 0.48 in males and 0.49 in females. The rates of central obesity at the cut-off point for WHtR of 0.50 were 43.6% in the whole population, 40.2% in males and 46.9% in females. All of these indicated that the prevalence of central obesity, at a WHtR of 0.50, was not low, especially among Chinese women, and thus more health promotion and health education efforts should focus on them.

Although the cut-off point of WHtR at 0.50 was certified effective for central obesity, it is still necessary to define a reasonable boundary, including an upper-boundary representing a severe level of obesity and CVD risks, to make those with high values of WHtR aware of the health risks they faced and encourage them to take action to control their weight and prevent CVD as soon as possible. A lower-boundary is also important to show that, as with BMI, lower is not always better in regard to WHtR. Above-low WHtR might be an indication of some nutrient deficiencies and increased risk of some other health issues. Ashwell [13–15] gave WHTR values of 0.40 and 0.60 to represent ‘underweight’ and ‘obvious central obesity’, respectively, based solely on the pragmatism principle. The method that yielded the range of 0.40 to 0.60 was scientifically deficient due to lack of data analysis. There is not currently sufficient evidence to definitively verify the boundary of WHtR for central obesity and CVD risks globally. As a result, we need to scientifically explore a boundary using data analysis from our large-scale population.

In general, we used specificity above 90% as a criterion, as with our previous study of WC, to determine the upper-boundary of an index by diagnosis method [29, 33]. **Table 2** lists the specificity at each point of WHtR to identify the upper-boundary in CVD risk factor clusters for severe obesity and CVD risk. Based on the results of the optimal upper boundary of WHtR and the 90^th^ percentile distribution of the WHtR, we considered WHtR of 0.57 as the optimal upper-boundary for severe obesity and CVD risk for the whole population, and among both genders, to ensure both efficacy and practical convenience.

When we use WHtR to assess central obesity level in practice, it does not mean that lower values of WHtR are better. If we do not define a reasonable lower boundary value of WHtR, most lay people will misunderstand and assume that blindly and excessively losing weight is acceptable, which would lead to health problems on the other end of the spectrum, including malnutrition. A meta-analysis of body mass index (BMI) in the Chinese population noted that the health risks caused by low BMI should also be a concern. The low BMI and high mortality in the Chinese population conformed to the cause of death profile in the Chinese population [30, 34]. The World Health Organization (WHO) recommended a lower boundary of BMI of 18.5 kg/m^2^ for underweight [31]. In **Table 5,** the lower boundary of WHtR is 0.40, indicating above underweight among the Chinese adults in the study. Our results, using a large-scale, population-based scientific method, were consistent with the reference point in Ashwell’s study [15], specifically a lower boundary of WHtR of 0.40. However, there is still somewhat of a lack of a relationship between WHtR and health status in the population, which requires further research.

According to the results of the study, we verified that a WHtR of 0.50 is an optimal cut-off point for central obesity diagnosis and CVD risk prediction in the Chinese adult population for both genders. We recommend the optimal boundaries of WHtR of 0.40 and 0.57, indicating underweight and severe central obesity, respectively, in Chinese adults. Compare with the BMI, We propose that WHtR might be a better predictor of CVD risk in the Chinese population for the following reasons: (1) WHtR is more highly correlated with visceral fat mass [32] and clustering of CVD risk factors in both children [33] and adults [34]; (2) WHtR may be a more accurate tracking indicator of fat distribution and accumulation by age because it accounts for the growth in both WC and height over age, particularly in children and adolescents [35]; and (3) the value of WHtR is free of measurement units and is similar among males and females at each age group, is easy to remember, and therefore has significant public health value. Finally, the new anthropometric index, WHtR, is appropriate for use in central obesity control, CVD prevention and public health promotion in China. The next step is to compare the efficacy of WHtR and WC in different height sub-groups for central obesity control and CVD risk prediction in the Chinese population.

There were several important limitations worth noting. First, the age distribution of the study population ranged from 35 to 70 years old and did not contain the entire Chinese population age distribution. Therefore, we cannot confirm that the cut-off point value and boundary values of WHtR can be used in younger and older populations. Second, the data used in the analysis was only baseline data. Follow-up and endpoint data would be better for exploring the cut-point value of WHtR as an indicator of CVD risk. Thus, further studies that relate anthropometric indices to clinical CVD mortality and all-cause death rate are needed.

## Conclusions

For the whole study population, the optimal WHtR cut-off point for the CVD risk factor cluster was 0.50. The cut-off points for severe central obesity were 0.57 in the whole population. The P_5_ of WHtR, which represents the lower boundary values of WHtR that indicates above underweight, was 0.40 in the whole population. WHtR 0.50 was an optimal cut-off point for evaluating CVD risks in Chinese adults of both genders. The optimal boundaries of WHtR were 0.40 and 0.57, indicating low body weight and severe risk for CVD, respectively, in Chinese adults.

## Supporting Information

S1 Dataset Analysis dataset. (SAV)

S1 Table Cut-off Point Values of WHtR for Predictive of Hypertension. Values are cut-off point values of WHtR in the first column, ROC least distances in the last column and percentage rates (%) in the other columns, which indicated some main diagnostic rate. Abbreviations see Tables 1 and 2.
(DOCX)

S2 Table Cut-off Point Values of WHtR for Predictive of Diabetes Mellitus Values are cut-off point values of WHtR in the first column, ROC least distances in the last column and percentage rates (%) in the other columns, which indicated some main diagnostic rate. Abbreviations see Tables 1 and 2.
(DOCX)

S3 Table Cut-off Point Values of WHtR for Predictive of High TG Values are cut-off point values of WHtR in the first column, ROC least distances in the last column and percentage rates (%) in the other columns, which indicated some main diagnostic rate. Abbreviations see Tables 1 and 2.
(DOCX)

S4 Table Cut-off Point Values of WHtR for Predictive of High LDL-C Values are cut-off point values of WHtR in the first column, ROC least distances in the last column and percentage rates (%) in the other columns, which indicated some main diagnostic rate. Abbreviations see Tables 1 and 2.
(DOCX)

S5 Table Cut-off Point Values of WHtR for Predictive of Low HDL-C Values are cut-off point values of WHtR in the first column, ROC least distances in the last column and percentage rates (%) in the other columns, which indicated some main diagnostic rate. Abbreviations see Tables 1 and 2.
(DOCX)
