# Correction: The Cut-Off Point and Boundary Values of Waist-to-Height Ratio as an Indicator for Cardiovascular Risk Factors in Chinese Adults from the PURE Study

**DOI:** 10.1371/journal.pone.0161551

**Published:** 2016-08-16

**Authors:** Yaguang Peng, Wei Li, Yang Wang, Jian Bo, Hui Chen

There are errors in Table 1. Please see the correct Table 1 and its caption here.

**Table 1:** Characteristics of the Study Chinese Population in the PURE Study.

	All Subjects	Male	Female
Number(cases)	43841	18019	25822
Age(year)	51.27±7.17	51.68±9.91	50.98±9.62
Height(cm)	160.79±8.25	167.42±6.48	156.15±5.83*
Weight(kg)	63.82±12.13	68.76±12.10	60.37±10.89*
WC(cm)	81.10±10.54	83.76±10.40	79.24±10.24*
WHtR	0.50±0.06	0.50±0.06	0.51±0.06*
BMI(m/kg^2^)	24.63±4.02	24.48±3.80	24.74±4.15*
SBP(mmHg)	133.56±22.42	135.08±21.09	132.51±23.25*
DBP(mmHg)	82.70±12.25	83.87±12.38	81.88±12.09*
FBG(mmol/L)	5.56±1.56	5.56±1.55	5.57±1.56
TG(mmol/L)	1.55±1.10	1.60±1.20	1.51±1.02
LDL-C(mmol/L)	2.63±0.79	2.56±0.76	2.67±0.81*
HDL-C(mmol/L)	1.36±0.33	1.32±0.33	1.39±0.33*
HP(%)	42.7	45.3	40.9*
DM(%)	9.2	9.3	9.1
High TG(%)	29.6	31.0	28.6*
High LDL-C(%)	23.4	20.6	25.4*
Low HDL-C(%)	17.9	12.2	14.6*
RFC(%)	35.4	37.1	34.2*

Values are means ± SD or n (%). * P<0.05.

* Values of female group are significantly different from those of male group.

P values are from t-tests or chi-square tests for analysis of variance for continuous variables and categorical variables.

WC, waist circumference; WHtR, waist-to-height ratio; SBP, systolic blood pressure; DBP, diastolic blood pressure; FBG, fasting blood glucose; TG, triglycerides; LDL-C, low-density lipoprotein cholesterol; HDL-C, high-density lipoprotein cholesterol; HP, hypertension; DM, diabetes mellitus; RFC, risk factors cluster.

## References

[1] Peng Y, Li W, Wang Y, Bo J, Chen H (2015) The Cut-Off Point and Boundary Values of Waist-to-Height Ratio as an Indicator for Cardiovascular Risk Factors in Chinese Adults from the PURE Study. PLoS ONE 10(12): e0144539 doi: 10.1371/journal.pone.0144539 26642201 10.1371/journal.pone.0144539 PMC4671670

