# Supplementary material for: The Cut-Off Point and Boundary Values of Waist-to-Height Ratio as an Indicator for Cardiovascular Risk Factors in Chinese Adults from the PURE Study
Source: PLoS One. 2015 Dec 7;10(12):e0144539. doi: 10.1371/journal.pone.0144539 (PMC4671670; doi:10.1371/journal.pone.0144539)
Supplement: S1 Table — Values are cut-off point values of WHtR in the first column, ROC least distances in the last column and percentage rates (%) in the other columns, which indicated some main diagnostic rate. Abbreviations see Tables 1 and 2. (DOCX) [file pone.0144539.s002.docx]

**S1 Table.** Cut-off Point Values of WHtR for Predictive of Hypertension

| Hypertension | Value | Sen. | Spe. | ROC Least Dis. |
| --- | --- | --- | --- | --- |
| All Subjects (n=43 841) | 0.48 | 0.742 | 0.489 | 0.572 |
|  | 0.49 | 0.685 | 0.554 | 0.546 |
|  | 0.50 | 0.625 | 0.619 | 0.535 |
|  | 0.51 | 0.559 | 0.679 | 0.545 |
|  | 0.52 | 0.493 | 0.736 | 0.572 |
|  | 0.53 | 0.426 | 0.787 | 0.612 |
|  | 0.54 | 0.364 | 0.828 | 0.659 |
|  | 0.55 | 0.305 | 0.866 | 0.708 |
|  | 0.56 | 0.253 | 0.898 | 0.754 |
|  | 0.57 | 0.207 | 0.921 | 0.797 |
|  | 0.58 | 0.168 | 0.940 | 0.834 |
|  | 0.59 | 0.133 | 0.955 | 0.868 |
|  | 0.60 | 0.103 | 0.967 | 0.898 |
| Male  (n=18 019) | 0.48 | 0.721 | 0.491 | 0.580 |
|  | 0.49 | 0.657 | 0.555 | 0.562 |
|  | 0.50 | 0.589 | 0.623 | 0.558 |
|  | 0.51 | 0.516 | 0.688 | 0.576 |
|  | 0.52 | 0.443 | 0.749 | 0.611 |
|  | 0.53 | 0.373 | 0.804 | 0.657 |
|  | 0.54 | 0.308 | 0.845 | 0.710 |
|  | 0.55 | 0.251 | 0.886 | 0.758 |
|  | 0.56 | 0.201 | 0.915 | 0.803 |
|  | 0.57 | 0.158 | 0.939 | 0.844 |
|  | 0.58 | 0.119 | 0.956 | 0.882 |
|  | 0.59 | 0.086 | 0.967 | 0.914 |
|  | 0.60 | 0.063 | 0.977 | 0.937 |
| Female (n=25 822) | 0.48 | 0.758 | 0.487 | 0.567 |
|  | 0.49 | 0.707 | 0.553 | 0.534 |
|  | 0.50 | 0.653 | 0.616 | 0.518 |
|  | 0.51 | 0.593 | 0.674 | 0.522 |
|  | 0.52 | 0.531 | 0.727 | 0.543 |
|  | 0.53 | 0.467 | 0.776 | 0.578 |
|  | 0.54 | 0.407 | 0.818 | 0.620 |
|  | 0.55 | 0.347 | 0.853 | 0.670 |
|  | 0.56 | 0.292 | 0.886 | 0.717 |
|  | 0.57 | 0.245 | 0.909 | 0.760 |
|  | 0.58 | 0.206 | 0.930 | 0.797 |
|  | 0.59 | 0.169 | 0.948 | 0.832 |
|  | 0.60 | 0.134 | 0.960 | 0.867 |

Values are cut-off point values of WHtR in the first column, ROC least distances in the last column and percentage rates (%) in the other columns, which indicated some main diagnostic rate.

Abbreviations see Table 1,2.
