# Supplementary material for: The Cut-Off Point and Boundary Values of Waist-to-Height Ratio as an Indicator for Cardiovascular Risk Factors in Chinese Adults from the PURE Study
Source: PLoS One. 2015 Dec 7;10(12):e0144539. doi: 10.1371/journal.pone.0144539 (PMC4671670; doi:10.1371/journal.pone.0144539)
Supplement: S2 Table — Abbreviations see Tables 1 and 2. (DOCX) [file pone.0144539.s003.docx]

**S2 Table B.** Cut-off Point Values of WHtR for Predictive of Diabetes Mellitus

| Diabetes | Value | Sen. | Spe. | ROC Least Dis. |
| --- | --- | --- | --- | --- |
| All Subjects (n=43 841) | 0.48 | 0.817 | 0.411 | 0.617 |
|  | 0.49 | 0.763 | 0.473 | 0.578 |
|  | 0.50 | 0.704 | 0.536 | 0.550 |
|  | 0.51 | 0.640 | 0.599 | 0.539 |
|  | 0.52 | 0.575 | 0.660 | 0.545 |
|  | 0.53 | 0.505 | 0.716 | 0.571 |
|  | 0.54 | 0.439 | 0.765 | 0.608 |
|  | 0.55 | 0.365 | 0.809 | 0.663 |
|  | 0.56 | 0.308 | 0.848 | 0.709 |
|  | 0.57 | 0.259 | 0.879 | 0.751 |
|  | 0.58 | 0.207 | 0.904 | 0.799 |
|  | 0.59 | 0.161 | 0.925 | 0.842 |
|  | 0.60 | 0.127 | 0.943 | 0.875 |
| Male  (n=18 019) | 0.48 | 0.793 | 0.414 | 0.621 |
|  | 0.49 | 0.736 | 0.479 | 0.585 |
|  | 0.50 | 0.669 | 0.547 | 0.561 |
|  | 0.51 | 0.589 | 0.615 | 0.563 |
|  | 0.52 | 0.517 | 0.680 | 0.579 |
|  | 0.53 | 0.440 | 0.741 | 0.617 |
|  | 0.54 | 0.370 | 0.791 | 0.664 |
|  | 0.55 | 0.294 | 0.836 | 0.725 |
|  | 0.56 | 0.239 | 0.873 | 0.772 |
|  | 0.57 | 0.193 | 0.904 | 0.813 |
|  | 0.58 | 0.144 | 0.929 | 0.859 |
|  | 0.59 | 0.106 | 0.948 | 0.895 |
|  | 0.60 | 0.081 | 0.963 | 0.920 |
| Female (n=25 822) | 0.48 | 0.834 | 0.409 | 0.614 |
|  | 0.49 | 0.783 | 0.470 | 0.573 |
|  | 0.50 | 0.728 | 0.529 | 0.544 |
|  | 0.51 | 0.676 | 0.588 | 0.524 |
|  | 0.52 | 0.616 | 0.645 | 0.523 |
|  | 0.53 | 0.551 | 0.699 | 0.540 |
|  | 0.54 | 0.489 | 0.747 | 0.570 |
|  | 0.55 | 0.417 | 0.790 | 0.620 |
|  | 0.56 | 0.357 | 0.830 | 0.665 |
|  | 0.57 | 0.306 | 0.861 | 0.707 |
|  | 0.58 | 0.252 | 0.887 | 0.757 |
|  | 0.59 | 0.201 | 0.910 | 0.804 |
|  | 0.60 | 0.160 | 0.930 | 0.842 |

Values are cut-off points of WHtR in the first column, ROC least distances in the last column and percentage rates (%) in the other columns, which indicated some main diagnostic rate.

Abbreviations see Table 1,2.
