# Supplementary material for: The Cut-Off Point and Boundary Values of Waist-to-Height Ratio as an Indicator for Cardiovascular Risk Factors in Chinese Adults from the PURE Study
Source: PLoS One. 2015 Dec 7;10(12):e0144539. doi: 10.1371/journal.pone.0144539 (PMC4671670; doi:10.1371/journal.pone.0144539)
Supplement: S3 Table — Abbreviations see Tables 1 and 2. (DOCX) [file pone.0144539.s004.docx]

**S3 Table.** Cut-off Point Values of WHtR for Predictive of High TG

| High TG | Value | Sen. | Spe. | ROC Least Dis. |
| --- | --- | --- | --- | --- |
| All Subjects (n=43 841) | 0.48 | 0.796 | 0.468 | 0.569 |
|  | 0.49 | 0.740 | 0.532 | 0.535 |
|  | 0.50 | 0.680 | 0.596 | 0.515 |
|  | 0.51 | 0.613 | 0.657 | 0.517 |
|  | 0.52 | 0.541 | 0.713 | 0.541 |
|  | 0.53 | 0.467 | 0.764 | 0.582 |
|  | 0.54 | 0.399 | 0.808 | 0.631 |
|  | 0.55 | 0.332 | 0.845 | 0.686 |
|  | 0.56 | 0.272 | 0.878 | 0.738 |
|  | 0.57 | 0.225 | 0.905 | 0.781 |
|  | 0.58 | 0.179 | 0.925 | 0.824 |
|  | 0.59 | 0.140 | 0.942 | 0.862 |
|  | 0.60 | 0.109 | 0.956 | 0.892 |
| Male  (n=18 019) | 0.48 | 0.801 | 0.483 | 0.554 |
|  | 0.49 | 0.741 | 0.549 | 0.520 |
|  | 0.50 | 0.673 | 0.617 | 0.504 |
|  | 0.51 | 0.597 | 0.683 | 0.513 |
|  | 0.52 | 0.516 | 0.742 | 0.549 |
|  | 0.53 | 0.432 | 0.794 | 0.604 |
|  | 0.54 | 0.359 | 0.837 | 0.661 |
|  | 0.55 | 0.287 | 0.874 | 0.724 |
|  | 0.56 | 0.229 | 0.904 | 0.777 |
|  | 0.57 | 0.181 | 0.929 | 0.822 |
|  | 0.58 | 0.135 | 0.947 | 0.866 |
|  | 0.59 | 0.097 | 0.961 | 0.904 |
|  | 0.60 | 0.070 | 0.972 | 0.930 |
| Female (n=25 822) | 0.48 | 0.792 | 0.458 | 0.580 |
|  | 0.49 | 0.739 | 0.521 | 0.545 |
|  | 0.50 | 0.685 | 0.582 | 0.523 |
|  | 0.51 | 0.624 | 0.640 | 0.520 |
|  | 0.52 | 0.561 | 0.694 | 0.535 |
|  | 0.53 | 0.494 | 0.745 | 0.567 |
|  | 0.54 | 0.430 | 0.788 | 0.608 |
|  | 0.55 | 0.365 | 0.826 | 0.658 |
|  | 0.56 | 0.304 | 0.860 | 0.710 |
|  | 0.57 | 0.258 | 0.888 | 0.750 |
|  | 0.58 | 0.212 | 0.909 | 0.793 |
|  | 0.59 | 0.173 | 0.929 | 0.830 |
|  | 0.60 | 0.139 | 0.946 | 0.862 |

Values are cut-off points of WHtR in the first column, ROC least distances in the last column and percentage rates (%) in the other columns, which indicated some main diagnostic rate.

Abbreviations see Table 1,2.
