# Supplementary material for: The Cut-Off Point and Boundary Values of Waist-to-Height Ratio as an Indicator for Cardiovascular Risk Factors in Chinese Adults from the PURE Study
Source: PLoS One. 2015 Dec 7;10(12):e0144539. doi: 10.1371/journal.pone.0144539 (PMC4671670; doi:10.1371/journal.pone.0144539)
Supplement: S4 Table — Abbreviations see Tables 1 and 2. (DOCX) [file pone.0144539.s005.docx]

**S4 Table.** Cut-off Point Values of WHtR for Predictive of High LDL-C

| High LDL-C | Value | Sen. | Spe. | ROC Least Dis. |
| --- | --- | --- | --- | --- |
| All Subjects (n=43 841) | 0.48 | 0.684 | 0.413 | 0.667 |
|  | 0.49 | 0.618 | 0.473 | 0.651 |
|  | 0.50 | 0.557 | 0.536 | 0.642 |
|  | 0.51 | 0.490 | 0.598 | 0.650 |
|  | 0.52 | 0.422 | 0.657 | 0.672 |
|  | 0.53 | 0.358 | 0.712 | 0.704 |
|  | 0.54 | 0.300 | 0.761 | 0.740 |
|  | 0.55 | 0.245 | 0.805 | 0.779 |
|  | 0.56 | 0.195 | 0.842 | 0.820 |
|  | 0.57 | 0.158 | 0.874 | 0.851 |
|  | 0.58 | 0.125 | 0.900 | 0.881 |
|  | 0.59 | 0.097 | 0.922 | 0.907 |
|  | 0.60 | 0.075 | 0.940 | 0.927 |
| Male  (n=18 019) | 0.48 | 0.691 | 0.417 | 0.659 |
|  | 0.49 | 0.619 | 0.479 | 0.646 |
|  | 0.50 | 0.548 | 0.546 | 0.640 |
|  | 0.51 | 0.473 | 0.613 | 0.654 |
|  | 0.52 | 0.397 | 0.678 | 0.684 |
|  | 0.53 | 0.326 | 0.737 | 0.723 |
|  | 0.54 | 0.265 | 0.787 | 0.765 |
|  | 0.55 | 0.204 | 0.831 | 0.813 |
|  | 0.56 | 0.159 | 0.868 | 0.852 |
|  | 0.57 | 0.123 | 0.900 | 0.883 |
|  | 0.58 | 0.092 | 0.925 | 0.911 |
|  | 0.59 | 0.065 | 0.945 | 0.936 |
|  | 0.60 | 0.048 | 0.960 | 0.953 |
| Female (n=25 822) | 0.48 | 0.680 | 0.410 | 0.671 |
|  | 0.49 | 0.618 | 0.469 | 0.654 |
|  | 0.50 | 0.561 | 0.529 | 0.644 |
|  | 0.51 | 0.500 | 0.586 | 0.649 |
|  | 0.52 | 0.437 | 0.641 | 0.668 |
|  | 0.53 | 0.375 | 0.694 | 0.695 |
|  | 0.54 | 0.320 | 0.741 | 0.728 |
|  | 0.55 | 0.269 | 0.785 | 0.762 |
|  | 0.56 | 0.216 | 0.823 | 0.804 |
|  | 0.57 | 0.178 | 0.854 | 0.835 |
|  | 0.58 | 0.144 | 0.881 | 0.865 |
|  | 0.59 | 0.114 | 0.905 | 0.891 |
|  | 0.60 | 0.091 | 0.926 | 0.912 |

Values are cut-off points of WHtR in the first column, ROC least distances in the last column and percentage rates (%) in the other columns, which indicated some main diagnostic rate.

Abbreviations see Table 1,2.
