# Supplementary material for: The Cut-Off Point and Boundary Values of Waist-to-Height Ratio as an Indicator for Cardiovascular Risk Factors in Chinese Adults from the PURE Study
Source: PLoS One. 2015 Dec 7;10(12):e0144539. doi: 10.1371/journal.pone.0144539 (PMC4671670; doi:10.1371/journal.pone.0144539)
Supplement: S5 Table — Abbreviations see Tables 1 and 2. (DOCX) [file pone.0144539.s006.docx]

**S5 Table.** Cut-off Point Values of WHtR for Predictive of Low HDL-C

| Low HDL-C | Value | Sen. | Spe. | ROC Least Dis. |
| --- | --- | --- | --- | --- |
| All Subjects (n=43 841) | 0.48 | 0.639 | 0.395 | 0.704 |
|  | 0.49 | 0.583 | 0.458 | 0.684 |
|  | 0.50 | 0.514 | 0.519 | 0.684 |
|  | 0.51 | 0.450 | 0.582 | 0.691 |
|  | 0.52 | 0.382 | 0.642 | 0.715 |
|  | 0.53 | 0.319 | 0.698 | 0.745 |
|  | 0.54 | 0.266 | 0.748 | 0.776 |
|  | 0.55 | 0.216 | 0.795 | 0.811 |
|  | 0.56 | 0.175 | 0.835 | 0.842 |
|  | 0.57 | 0.138 | 0.867 | 0.873 |
|  | 0.58 | 0.108 | 0.894 | 0.898 |
|  | 0.59 | 0.081 | 0.917 | 0.923 |
|  | 0.60 | 0.063 | 0.937 | 0.939 |
| Male  (n=18 019) | 0.48 | 0.667 | 0.409 | 0.679 |
|  | 0.49 | 0.607 | 0.473 | 0.658 |
|  | 0.50 | 0.531 | 0.539 | 0.657 |
|  | 0.51 | 0.457 | 0.607 | 0.671 |
|  | 0.52 | 0.374 | 0.670 | 0.708 |
|  | 0.53 | 0.306 | 0.730 | 0.744 |
|  | 0.54 | 0.251 | 0.782 | 0.781 |
|  | 0.55 | 0.202 | 0.829 | 0.816 |
|  | 0.56 | 0.164 | 0.868 | 0.846 |
|  | 0.57 | 0.127 | 0.900 | 0.879 |
|  | 0.58 | 0.095 | 0.925 | 0.908 |
|  | 0.59 | 0.071 | 0.946 | 0.931 |
|  | 0.60 | 0.049 | 0.960 | 0.952 |
| Female (n=25 822) | 0.48 | 0.610 | 0.387 | 0.727 |
|  | 0.49 | 0.558 | 0.447 | 0.707 |
|  | 0.50 | 0.496 | 0.506 | 0.705 |
|  | 0.51 | 0.444 | 0.566 | 0.706 |
|  | 0.52 | 0.390 | 0.623 | 0.717 |
|  | 0.53 | 0.332 | 0.678 | 0.742 |
|  | 0.54 | 0.282 | 0.727 | 0.768 |
|  | 0.55 | 0.230 | 0.772 | 0.803 |
|  | 0.56 | 0.185 | 0.813 | 0.836 |
|  | 0.57 | 0.149 | 0.845 | 0.865 |
|  | 0.58 | 0.121 | 0.874 | 0.888 |
|  | 0.59 | 0.092 | 0.899 | 0.914 |
|  | 0.60 | 0.077 | 0.921 | 0.926 |

Values are cut-off points of WHtR in the first column, ROC least distances in the last column and percentage rates (%) in the other columns, which indicated some main diagnostic rate.

Abbreviations see Table 1,2.
